# Enhanced droplet collision rates and impact velocities in turbulent flows: The effect of poly-dispersity and transient phases

**DOI:** 10.1038/s41598-017-12093-0

**Published:** 2017-09-25

**Authors:** Martin James, Samriddhi Sankar Ray

**Affiliations:** 1 Max Planck Institute for Dynamics and Self-Organization (MPI DS), Am Faßberg 17, 37077 Göttingen, Germany; 2 Department of Physics, Indian Institute of Science, Bangalore, 560012 India; 3 International Centre for Theoretical Sciences, Tata Institute of Fundamental Research, Bangalore, 560089 India

## Abstract

We compare the collision rates and the typical collisional velocities amongst droplets of different sizes in a poly-disperse suspension advected by two- and three-dimensional turbulent flows. We show that the collision rate is enhanced in the transient phase for droplets for which the size-ratios between the colliding pairs is large as well as obtain precise theoretical estimates of the dependence of the impact velocity of particles-pairs on their relative sizes. These analytical results are validated against data from our direct numerical simulations. Our results suggest that an explanation of the rapid growth of droplets, e.g., in warm clouds, may well lie in the dynamics of particles in transient phases where increased collision rates between large and small particles could result in runaway process. Our results are also important to model coalescence or fragmentation (depending on the impact velocities) and will be crucial, for example, in obtaining precise coalescence kernels in such systems.

## Introduction

The study of collisions and coalescences of droplets in a turbulent flow is central to understanding a plethora of natural phenomena. Such phenomena, which span a variety of spatial and temporal scales, range from planet-formation in circumstellar disks^1,2^ to rain drop formation in warm clouds^3–6^. In particular, for the latter, the observed rapid growth of droplets, leading to rain, in a warm cloud is not yet completely understood. A common, key question underlying all such problems is to understand and explain the rate with which such droplets grow, essentially through collisions resulting in coalescences. In recent years as studies in turbulent transport and the dynamics of inertial particles have been at the forefront of research in fluid dynamics, there have been great advances in enunciating the role of turbulent mixing, preferential concentration and intermittency in the rapid growth of droplet sizes, through coalescences, in suspensions advected by a turbulent flow. Hence several questions related to the relative velocities and collision rates of droplets^4,7–16^, the enhanced settling in the presence of gravity^17–21^, and most recently the abrupt growth in droplet sizes through coalescence^22^ have been explored, theoretically and in experiments, in great detail. It is therefore of great importance in the area of the earth sciences as well as, more generally, in the context of non-equilibrium statistical mechanics to understand possible origins of this discrepancy in numerical and theoretical results from observed data^23^.

We know that the nucleation sites (aerosols) lead to condensation in the super-saturated environment of a warm cloud. However such a process leads to droplets which are around 8 *μm*, much smaller than the typical droplets sizes which initiate rain. It is believed that the turbulent airflow results in efficient mixing, coupled with the preferential concentration of such droplets, exactly similar to heavy, inertial particles in turbulence, yielding more and more collisions, often resulting in coalescences, to generate bigger and bigger droplets. Much of the work in this field have dealt with mono-disperse (same-sized droplets) suspensions in flows where both the particle dynamics and the turbulent flow itself is in a statistically stationary regime. Indeed it is now well established that turbulence enhances collision rates amongst droplets and could partially explain accelerated growth rates, e.g., of water droplets in clouds, which cannot be captured through kinetic models. However the bottleneck in estimating the early time initiation of rain–as observed in nature–is still an unresolved issue despite intense numerical and theoretical studies over the past few years^23^.

Much of the work related to the issues of collision rates and relative velocities of particle-pairs deal with systems of identical particles^21,24,25^. However in nature, the distribution of particles is typically inhomogeneous. Hence particles of different sizes interact with one another. Furthermore, to critically understand if coalescences dominate–leading to the growth of larger and larger droplets–an estimate of the strength of impact velocities of particles with different radii is crucial. In particular the role of preferential concentration (which changes with particle radii) of particles at small scales as well as possible large velocity differences due to caustics^8^ and the sling effect^4^ ought to result in non-trivial collisional velocities in an inhomogeneous size distribution of particles in a turbulent flow.

In this work, we study the complementary problems of the velocity difference between colliding droplets in two and three dimensions as well as the rate of collisions in such systems. Our approach is different from previous studies in two critical aspects: (1) Typically, earlier studies have focussed on a mono-disperse suspension of particles; we now look at a poly-disperse suspension of droplets. (2) Secondly, and more crucially, we also examine the collision, and hence coalescence, rates between different-sized particles not in the statistically steady state of particle dynamics but in the transient phase before the distribution converges to a non-equilibrium steady measure. Finally we measure the typical velocities of impacts–in the steady state–and obtain asymptotic scaling relations which are validated by our numerical simulation in two and three dimensions. To our knowledge this is the first time that the transient phase has been studied and, as we show below, our results indicate that reasons for the rapid growth of droplets seen in nature lie in this regime.

We focus on the aspects mentioned above because the collision frequency and the collision velocities are both key ingredients which ought to determine the growth of droplets. In nature, particle suspensions are inhomogeneous in their size distributions and far from the model mono-disperse suspension typically studied. Furthermore, in natural settings processes such as nucleation and droplet-droplet interactions, must be characterised by non-stationary (transient) measures, at least on short time scales. Indeed because of coalescences and condensation, the distribution of particle sizes are time-dependent and hence the dynamics of the droplet suspension rarely get a chance to converge to a non-equilibrium stationary state. It is natural, therefore, that such a problem is best studied within the framework of the transient phase of particle dynamics. Hence it behooves us to explore, numerically, the intriguing possibility of a further enhancement in collision rates in transient regimes as well as the possibility of accelerated droplet growths when the suspension itself is poly-disperse. We should emphasize that our turbulent advecting flow is in a non-equilibrium statistical steady state; the transients we refer to has to do with the transient, particle dynamics before they converge to their statistically steady measure; it is these transient phases which, for reasons explained above, are ubiquitous in nature and thus deserving of our attention.

For clarity, we first report our results on the impact velocities and then on collision frequencies in the section after that. We keep in mind that our results for the collisional velocities are for both two and three dimensions and are consistent with our analytical predictions. We discuss the issue of collision frequencies only in the context of two-dimensional flows; our preliminary results for 3D are consistent with the data reported here. However we will report the results on the collision frequencies in three dimensions, with a systematic in Froude and Stokes numbers, in future work.

## Results

We consider a fluid flow whose velocity ***u*** is a solution to the incompressible Navier–Stokes equation

1 $${\partial }_{t}{\boldsymbol{u}}+({\boldsymbol{u}}\cdot \nabla ){\boldsymbol{u}}=-\nabla p+\nu {\nabla }^{2}{\boldsymbol{u}}+{\boldsymbol{f}},\quad \nabla \cdot {\boldsymbol{u}}=0,$$

where *ν* designates the fluid kinematic viscosity. In two dimensions (2D), it is often convenient to re-write this in the vorticity (*ω*)-stream function (*ψ*) formulation^26,27^ as

2 $${\partial }_{t}\omega -J(\psi ,\omega )=\nu {\nabla }^{2}\omega +{f}_{\omega }-\mu \omega ,$$

where *J*(*ψ*, *ω*) ≡ (∂_*x*_ *ψ*) (∂_*y*_ *ω*) − (∂_*x*_ *ω*) (∂_*y*_ *ψ*) and *μ* is the coefficient of Ekman friction. At the point (*x*, *y*) the velocity ***u*** ≡ (−∂_*y*_ *ψ*, ∂_*x*_ *ψ*) and the vorticity *ω* = ∇*ψ*.

We now study the dynamics of small inertial particles (droplets) which are suspended in a turbulent flow field obtained as a solution of Eq. (1) in three dimensions (3D) or of Eq. (2) in two dimensions (2D) in the limit of small *ν* or large Reynolds numbers. We assume that our particles are much smaller than the Kolmogorov scale *η*, much heavier than the surrounding fluid, and with a small Reynolds number associated to their slip velocity. The motion of the i-th particle, in the turbulent fluid, is damped through a viscous Stokes drag and their trajectories ***x***_i_(*t*) are defined via

3 $$\frac{{\rm{d}}{{\boldsymbol{x}}}_{{\rm{i}}}}{{\rm{d}}t}={{\boldsymbol{v}}}_{{\rm{i}}},\quad \frac{{\rm{d}}{{\boldsymbol{v}}}_{{\rm{i}}}}{{\rm{d}}t}=-\frac{1}{{\tau }_{{\rm{p}}}}[{{\boldsymbol{v}}}_{{\rm{i}}}-{\boldsymbol{u}}({{\boldsymbol{x}}}_{{\rm{i}}},t)].$$

The relaxation time *τ*_p_ = 2*ρ*_p_ *a* ^2^/(9*ρ*_f_ *ν*), where *ρ*_p_ and *ρ*_f_ are the particle and fluid mass density respectively and *a* the particle radius, allows us to define a non-dimensional Stokes number *St* = *τ*_*p*_/*τ*_*η*_; the small time-scale *τ*_*η*_ is the Kolmogorov time scale and an intrinsic property of the fluid. In 2D, a natural Kolmogorov time scale is hard to define; we therefore measure the Stokes number *St* = *λ*_1_ *τ*_*p*_, where *λ*_1_ is the Lyapunov exponent of the flow. As was shown by in ref.^28^, this can be related to the smallest time-scale *τ*_min_ in 2D flows as the minimum value of ${(\sqrt{{k}^{3}E(k)})}^{-1}$. We obtain the radius of the particles from the definition of the Stokes time *τ*_*p*_ by choosing the density ratio to be 1000 (such as water in air).

### Impact Velocities

The impact velocity between colliding droplets, which determines the chance of coalescence or fragmentation, is defined as the velocity difference of the two particles at vanishing separation. Thence, we define the impact velocity between two particles, labelled 1 and 2, of Stokes numbers *St*_1_ and *St*_2_ as **Δ** = 〈|(**v**_2_ − **v**_1_) · $\hat{{\bf{r}}}$|〉, where the unit vector $\hat{{\bf{r}}}$ defines the vector connecting the centers of the two particles, with the constraint that (v_2_ − v_1_) · $\hat{{\bf{r}}}$ < 0 which define a pair of approaching particles (We note that the same definition can be arrived at by considering the velocity longitudinal structure function calculated in the limit where the pair separation goes to 0). The averaging 〈·〉 is defined over all colliding pairs.

**Figure 1:**
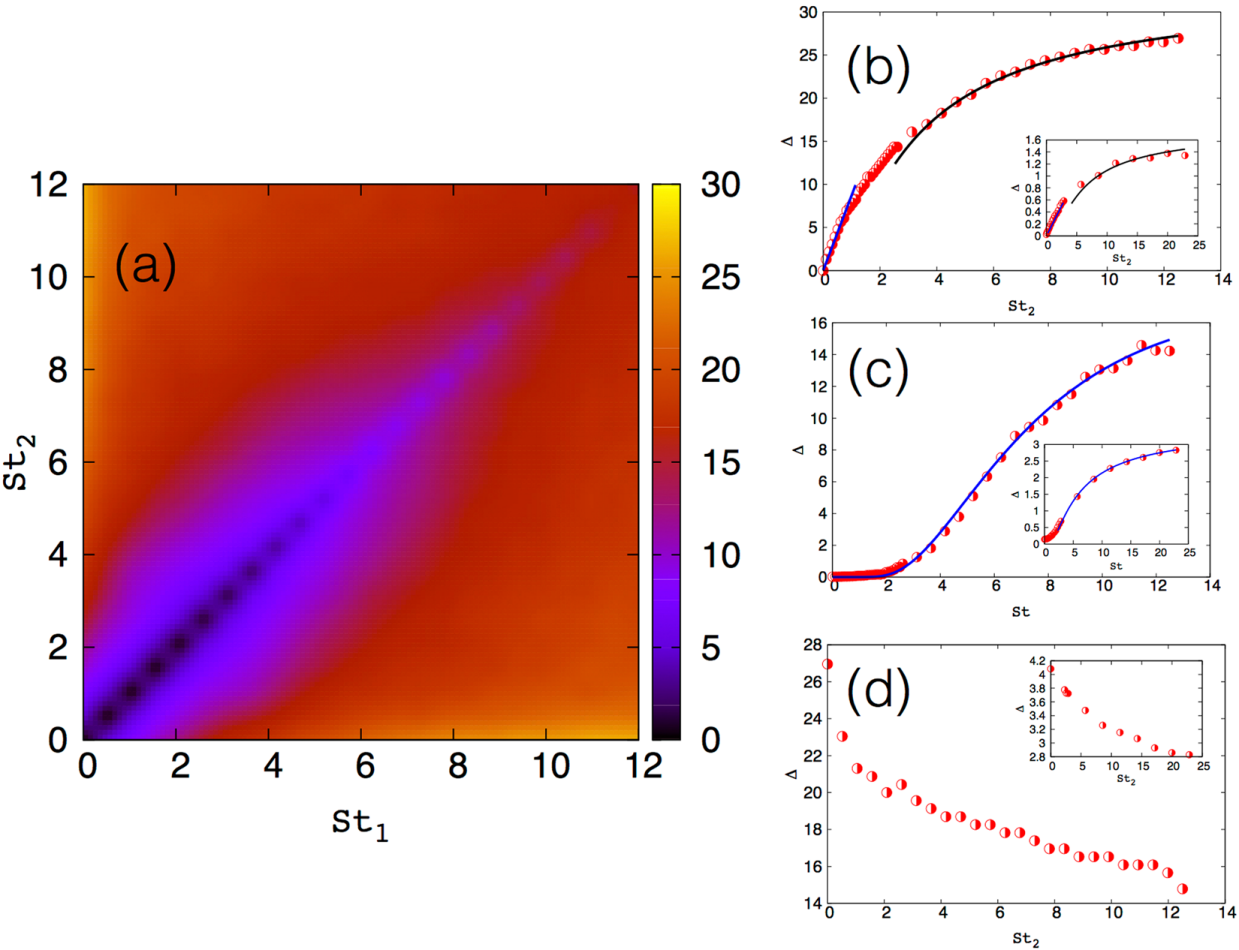
Impact velocities. (**a**) Impact velocity **Δ**(*St*_1_, *St*_2_) as a function of Stokes numbers of approaching particles in 2D. Representative plots of **Δ** as a function of (**b**) *St* for same-sized particles (**c**) *St*_2_ for $St=0.005\ll 1$ and for tracers in 3D, and (d) $St=12.5\gg 1$ in 2D and for $St=20\gg 1$ in 3D (inset). The blue solid curves (and black solid curve in (**b**)) are our theoretical predictions and the symbols are data from our simulations in 2D and 3D (inset). The error bars on our numerical data are comparable to the symbol size.

In Fig. 1(a) we show a pseudo-color plot of the amplitude of the impact velocity **Δ** as a function of the Stokes numbers of the colliding particles is shown from our data from the 2D simulations, with similar results obtained in our 3D DNS. (In this plot and all subsequent plots, we show **Δ** normalised by the Kolmogorov velocity *u*_*η*_ = *η*/*τ*_*η*_ of the underlying turbulent fluid; in 2D we choose *u*_*η*_ = *η*/*τ*_min_). Qualitatively, it is easy to understand the diagonal (*St*_1_ = *St*_2_) behaviour of **Δ**: The two-particle velocity correlation between particles (when the size of one particle is fixed and the other varied) attains a maximum when particles are of the same size (*St*_1_ = *St*_2_)^29^. Consequently, **Δ** attains a minimum when the approaching particles have the same Stokes number as is clearly seen in Fig. 1(a). For larger Stokes numbers, **Δ** becomes larger because of the formation of caustics which allow same-sized particles to collide with each other with arbitrarily large velocities.

In order to gain a complete understanding of the dependence of **Δ** on *St*_1_ and *St*_2_, it is useful to return to Eq. (3). Let us consider two particles 1 and 2 with Stokes times *τ*_1_ = *τ* and *τ*_2_ = *ατ*_1_ = *ατ*. We consider the non-dimensional form of Eq. (3) (by including factors of *τ*_*η*_) to obtain

$$\frac{d{{\bf{v}}}_{1}}{d\tau }=-\frac{1}{St}[{{\bf{v}}}_{1}-{{\bf{u}}}_{1}];$$

$$\frac{d{{\bf{v}}}_{2}}{d\tau }=-\frac{1}{\alpha St}[{{\bf{v}}}_{2}-{{\bf{u}}}_{2}].$$

For brevity, we set ***u***(***x***_i_, *t*) = **u**_i_. Thence we obtain

4 $$\frac{d{\boldsymbol{\Delta }}}{dt}=-\frac{1}{\alpha St}[{\boldsymbol{\Delta }}-St\mathrm{(1}-\alpha )\frac{d{{\bf{v}}}_{1}}{dt}\cdot \hat{{\bf{r}}}].$$

We have assumed here that at small particle separations |**r**|, the fluid velocity is smooth and hence $|{{\bf{u}}}_{1}-{{\bf{u}}}_{2}|\equiv \sigma \cdot {\bf{r}}\sim 0$, as |**r**| → 0 (*σ* is the gradient of the fluid velocity).

So far we have made only one defensible assumption in deriving Eq. (4) which has to do with the smoothness of the velocity field at small scales. To this extent Eq. (4) is exact in both 2D and 3D. Let us now explore the various asymptotics of this equation and obtain theoretical estimates of **Δ** for different combinations of Stokes numbers which can then be tested against data from our DNSs in 2D and 3D. The following limits naturally arise in this case (see Table 1 for a compact version of these limits): Case 1: *α* = 1 with no assumption on *St*; Case 2: Particle 1 is very small and close to being a tracer ($St\ll 1$) and $\alpha \sim {\mathscr{O}}\mathrm{(1)}$ such that $S{t}_{2}\mathop{ < }\limits_{ \tilde {}}1$; Case 3: Particle 1 is still small ($St\ll 1$) but $\alpha \gg 1$ such that $S{t}_{2}\gg 1$; and Case 4: For $St\mathop{ > }\limits_{ \tilde {}}1$. To obtain the limiting form of **Δ** from Eq. (4) in each such case, we assume that at time *t* = 0 the particles have come close to each other (without actually colliding) with a velocity difference **Δ**_0_ and then, over a time *τ*, they touch.

**Table 1:** Analytical Predictions.

Case	*St*_1_	*St*_2_	Prediction	Figure
Case 1	–	*St*_1_	**Δ** = **Δ**_0_ exp (−*τ*/*St*)	Fig. 1(b)
Case 2	$S{t}_{1}\ll 1$	$S{t}_{2}\mathop{ < }\limits_{ \tilde {}}1$	**Δ** ~ *St*_2_	Fig. 1(c)
Case 3	$S{t}_{1}\ll 1$	$S{t}_{2}\gg 1$	Δ = Δ_0_ exp (−*τ*/*St*_2_)	Fig. 1(c)
Case 4	$S{t}_{1}\mathop{ > }\limits_{ \tilde {}}1$	*St*_2_ ≠ *St*_1_	None	Fig. 1(d)

A summary of the different asymptotics and the theoretical predictions for **Δ**.

For *α* = 1 (Case 1), integrating Eq. (4), we obtain **Δ** = **Δ**_0_ exp (−*τ*/*St*), where (and in what follows) **Δ**_0_ is the constant of integration. In Fig. 1(b) we test our theoretical prediction (solid line) against data from our DNSs (symbols) in both 2D and 3D (inset) and find excellent agreement between the two.

We now address the important question of what happens when the two colliding particles have different Stokes numbers. Let us begin with Case 2 where $St\ll 1$ (*v*_1_ ≈ *u*_1_) and $\alpha \sim {\mathscr{O}}\mathrm{(1)}$. In this limit, we can rewrite Eq. (4) as

5 $$\alpha St\frac{d{\boldsymbol{\Delta }}}{dt}=-[{\boldsymbol{\Delta }}-St\mathrm{(1}-\alpha )\frac{d{{\bf{v}}}_{1}}{dt}\cdot \hat{{\bf{r}}}].$$

Since *αSt* < 1, we can set $\alpha St\frac{d{\boldsymbol{\Delta }}}{dt}=0$ and obtain ${\boldsymbol{\Delta }}\sim S{t}_{2}$. In the other limit, Case 3, where $St\ll 1$ and $\alpha \gg 1$ such that *αSt* > 1, we notice that $\frac{St\mathrm{(1}-\alpha )}{\alpha St}\frac{d{{\bf{v}}}_{1}}{dt}\cdot \hat{{\bf{r}}}\sim \frac{d{{\bf{v}}}_{1}}{dt}\cdot \hat{{\bf{r}}}\sim 0$ to leading order since $St\ll 1$. Thence we obtain **Δ** = **Δ**_0_ exp (−*τ*/*St*_2_).

Given the strong assumptions made in arriving at the two limits above, it is important to check our prediction against data from our DNSs. In Fig. 1(c) we show a representative plot of the impact velocity **Δ** between particles of Stokes number *St* = 0.005 (2D) and tracers (3D, inset) (For the 3D simulations, we choose tracers for the reference particle; our theoretical predictions can be shown to hold in the case when *St*_1_ = 0 as well) with particles of different Stokes numbers. We immediately notice that when $S{t}_{2}\mathop{ < }\limits_{ \tilde {}}1$, **Δ** is indeed linear with *St*_2_ and the data (symbols) consistent with our theoretical prediction shown as a blue curve. In the other limit when $S{t}_{2}\gg 1$, our data from numerical simulations is in excellent agreement with the theoretical prediction **Δ** ~ exp(−1/*St*_2_).

Let us finally turn to the situation when $St\mathop{ > }\limits_{ \tilde {}}1$ (Case 4). In Fig. 1(d) we show a representative plot of the impact velocity between a particle of Stokes number *St* = 12.5 (2D) and *St* = 20 (3D, inset) with all other particles. From our numerical data we see that that **Δ** shows rapid variation when $S{t}_{2}\mathop{ < }\limits_{ \tilde {}}S{t}_{1}$. However we do not have a self-consistent understanding of the functional form of the decrease in **Δ** from Eq. (4).

### Collision Rates

We now investigate the possibility of enhanced collision rates in turbulent flows. It is important to note that this work proposes a novel mechanism to explain a puzzle in the atmospheric sciences: Hence two-dimensional flows are useful for this study as it allows us, numerically, to study the collision rates in greater detail. This is in addition to the reason that atmospheric flows are often effectively modeled by the two-dimensional Navier-Stokes equation. We thus consider a poly-disperse suspension of droplets in a two-dimensional turbulent flow.

**Figure 2:**
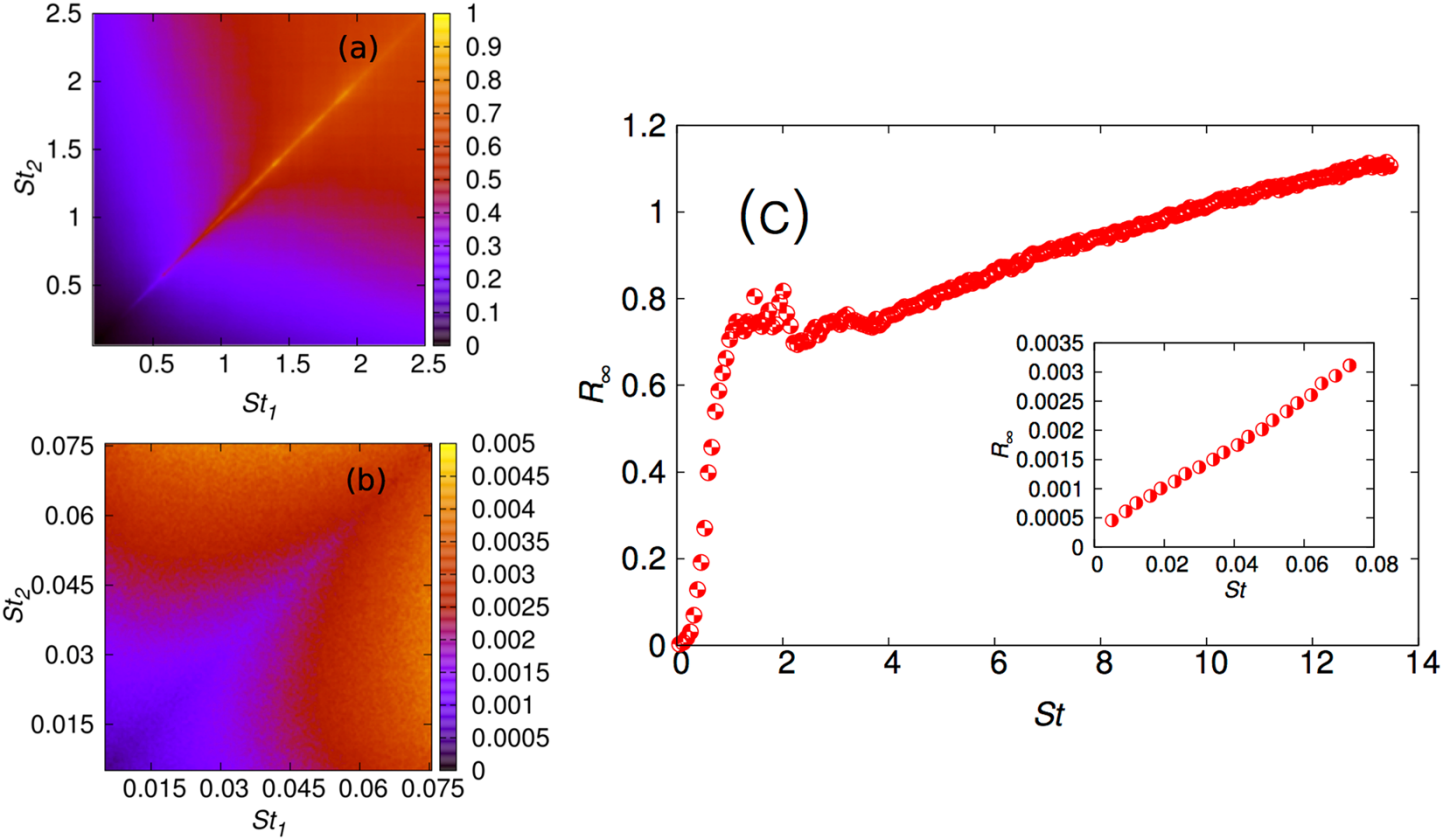
The collision rate *R*_∞_, in the steady state. (**a**) Pseudo-color plot of the collision rate *R*_∞_ as a function of *St*_1_ and *St*_2_ of the colliding pairs. In (**b**) we magnify the behaviour of *R*_∞_ for *St*_1_, $S{t}_{2}\ll 1$. The color-bar is normalised by the maximum in *R*_∞_ for the full Stokes number range. (**c**) Collision rate *R*_∞_ for collision between particles of the same size. In the inset we magnify the behaviour of *R*_∞_ in the small Stokes limit.

We begin by examining the collision rates *R*_∞_, in the statistical steady state, amongst particle-pairs of different Stokes numbers. In Fig. 2(a) we show the collision rate *R*_∞_, as a function of the Stokes numbers of the colliding pairs, in a pseudo-color plot in the statistically steady state. We see clearly a peak in *R*_∞_ for same-sized particles, i.e., along the diagonal in Fig. 2(a) since same-sized particles cluster much more than different-sized ones do. However, at very small Stokes numbers, in the limit of tracers, the collision rate shows a different behaviour (Fig. 2(b)) as has also been studied earlier for a smooth random flow by Bec, *et al*.^24^. In this tracer limit, particles are uniformly distributed which make collisions amongst particles of the same size less probable. By contrast, the higher probability of collisions among different-sized particles results from the finite impact velocity.

In order to illustrate this better, in Fig. 2(c) we show *R*_∞_ for same-sized particles as a function of their Stokes number. Given our definition of *R*_∞_, the Saffman-Turner prediction^30,31^ suggests a linear behaviour for two-dimensional flows for small Stokes numbers; in the inset of Fig. 2(c) we find an excellent agreement with this prediction. Our results in the large Stokes limit (Fig. 2(c)) suggests that, asymptotically, *R*_∞_ saturates to a constant in agreement with the Abrahamson limit^24,32^. We note that our collision rates, when considering same-sized particles in two-dimensional flow, are in complete agreement with previous theoretical and numerical studies for smooth random as well as turbulent flows in two dimensions^24,33^. We also observe the curiously sharp increase, with a peak for $St\mathop{ < }\limits_{ \tilde {}}1$, in the collision rate for same-sized particles which can be understood through models using a combination of clustering and caustics^23^.

**Figure 3:**
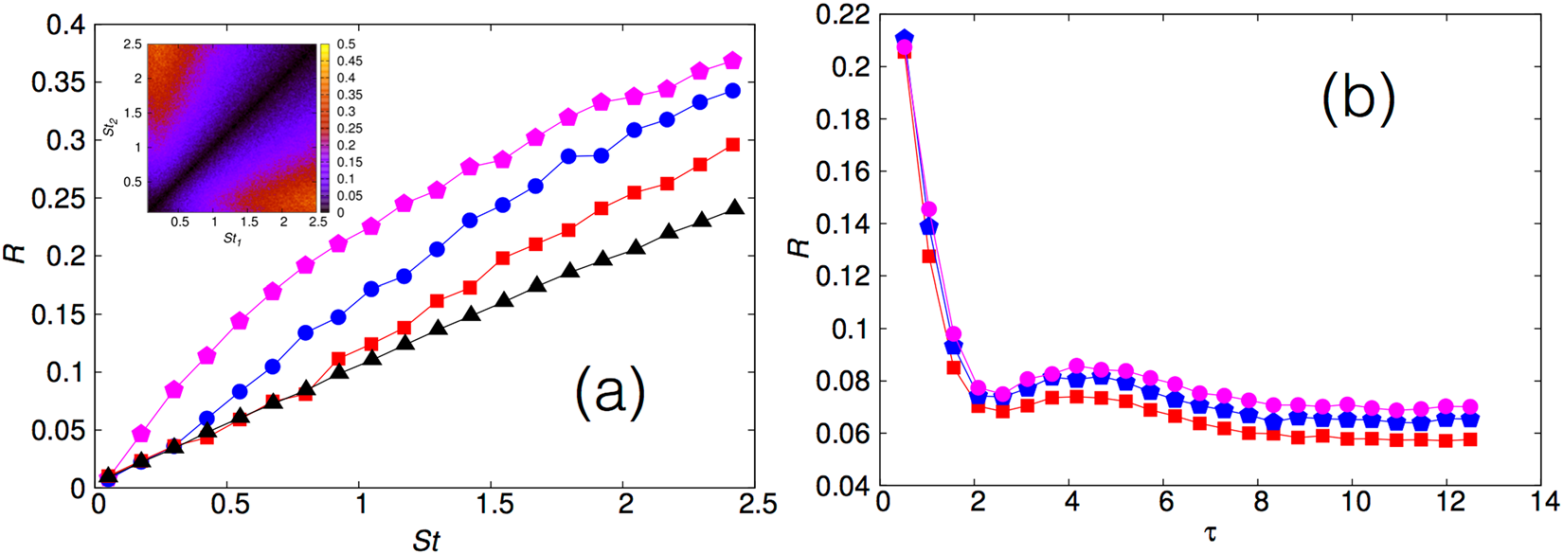
The collision rate *R*, in the transient phase. (**a**) The collision rate *R*, during the transient phase, between a reference particle with *St* = 0.05 and all the other particles. The curves correspond to times *t* = 0.5 *τ*_*η*_ (pink diamonds, uppermost curve), *t* = *τ*_*η*_ (blue circles) and *t* = 1.5 *τ*_*η*_ (red squares). The almost linear line with black triangles correspond the steady state distribution *R*_∞_ result. Inset: Pseudo color plot of the collision rate *R* during the transient phase; the color bar is normalised with the maximum in *R*_∞_. This snapshot is taken at time of the order of *τ*_*η*_. (**b**) The collision rate *R* as a function of time *τ* (normalised by *τ*_*η*_) for different-sized particles. We show representative plots for the case when *St*_1_ = 0.96 and for (i) *St*_2_ = 0.053 (pink circles), (ii) *St*_2_ = 0.03 (blue diamonds), and (iii) *St*_2_ = 0.005 (red squares). We observe a rapid decrease in the collision rate with time till it saturates to the asymptotic value *R*_∞_ at large times.

Let us now address the issue of collision rates *R* in the transient phase. It is worth reiterating that the advecting turbulent flow is in a non-equilibrium steady state: it is the particles which are in a non-stationary distribution and hence in a transient phase. In Fig. 3(a) (inset) we show *R* as a function of *St*_1_ and *St*_2_. We immediately note that in the transient phase there is an *enhanced* collision rate amongst particle pairs such that $S{t}_{1}\ll 1$ and $S{t}_{2}\gg 1$. This striking result shows that the chance to form bigger, or super drops, through the merging of large and small particles is accentuated in the transient phase and could be key to explaining the emergence of fat tails in droplet size distribution in a turbulent suspension, such as in warm clouds. In contrast, for colliding pairs with similar Stokes numbers and sizes, the collision rate is actually smaller in the transient phase when compared to the steady state distribution (Fig. 3(a), inset). This is easily understood in terms of the negative divergence of the velocity field of inertial particles leading to preferential concentration as *t* → ∞.

In order to illustrate this better, we take a reference particle in the small Stokes limit and measure the collision rate *R* with other particles of different Stokes numbers. In Fig. 3(a), we show a representative plot with the reference Stokes number *St* = 0.05 at different times. The lowest, linear curve is at the largest times when *R* → *R*_∞_. At smaller times, when the particle dynamics have not converged to the steady state distribution we find an increased collision rate as compared to the steady state distribution. Thus we have obtained strong numerical evidence for the enhancement of collision rates amongst dissimilar particles in the transient phase and the opposite effect for particles with the same Stokes number. It is now important to examine and characterise how *R* relaxes to *R*_∞_ in time. In Fig. 3(b) we show, for representative values of Stokes number, the approach to *R*_∞_ for different-Stokes particles. We observe a rapid decrease in *R* as a function of the normalised time *τ* = *t*/*τ*_min_ till it saturates to *R*_∞_. We also observe in this time-dependence a gentle oscillatory behaviour. Thence we characterise this relaxation process which illustrate that indeed for short, transient times there is significant enhancement of collision rates when compared to measurements made in the asymptotic steady state. A quantitative understanding of the functional form of this relaxation will be addressed elsewhere. It is clear from these plots that particles with smaller Stokes numbers tend to relax faster to their stationary distribution than the ones with larger Stokes number. In particular this relaxation time is typically, as seen from our plot, little larger than the shortest time-scale of the flow.

**Figure 4:**
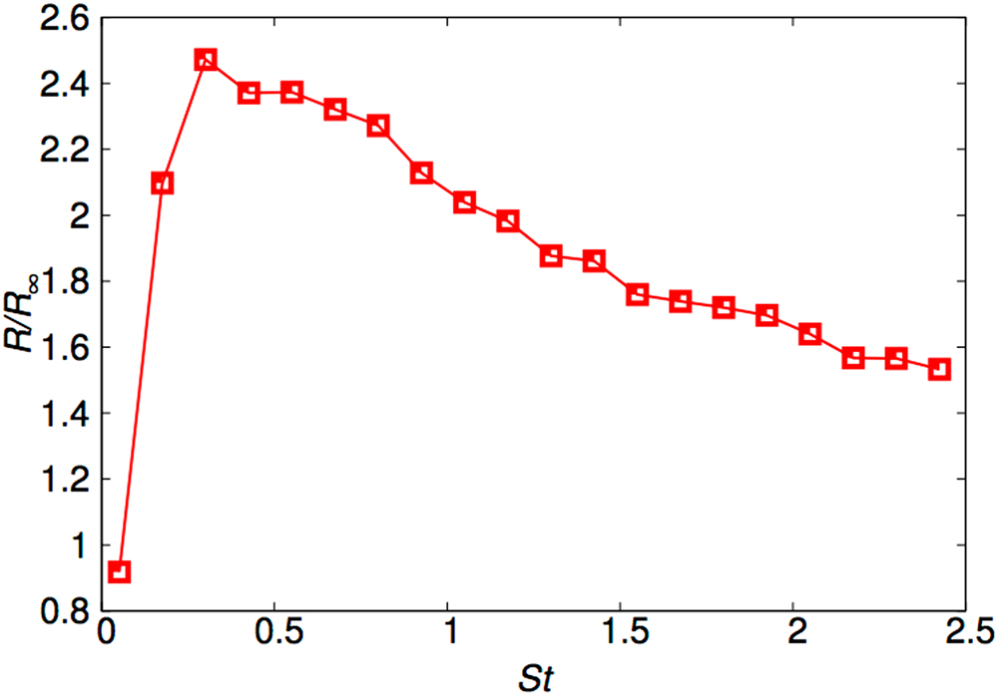
Enhancement factor. The enhancement factor *R*/*R*_∞_ for a reference particle with *St* = 0.05 in the transient phase (*t* = *τ*_*η*_).

Let us finally measure the actual enhancement of the collisions–and hence, coalescences–in the transient phase. This is quantified by taking the ratio *R*/*R*_∞_ and plotting it as a function of the Stokes number as shown in Fig. 4. In Fig. 4 we find a maximum enhancement of close to a factor of 2.5 for Stokes numbers ${\mathscr{O}}\mathrm{(1)}$ when it collides with a tracer-like (*St* = 0.05 in this case) particle in the transient phase as compared to the steady, long time distribution regime. We have looked at data with other small Stokes reference particle and found a similar behaviour as is suggested in Fig. 3(a) along and close to either the *St*_1_ or *St*_2_ axes.

## Discussion

**Figure 5:**
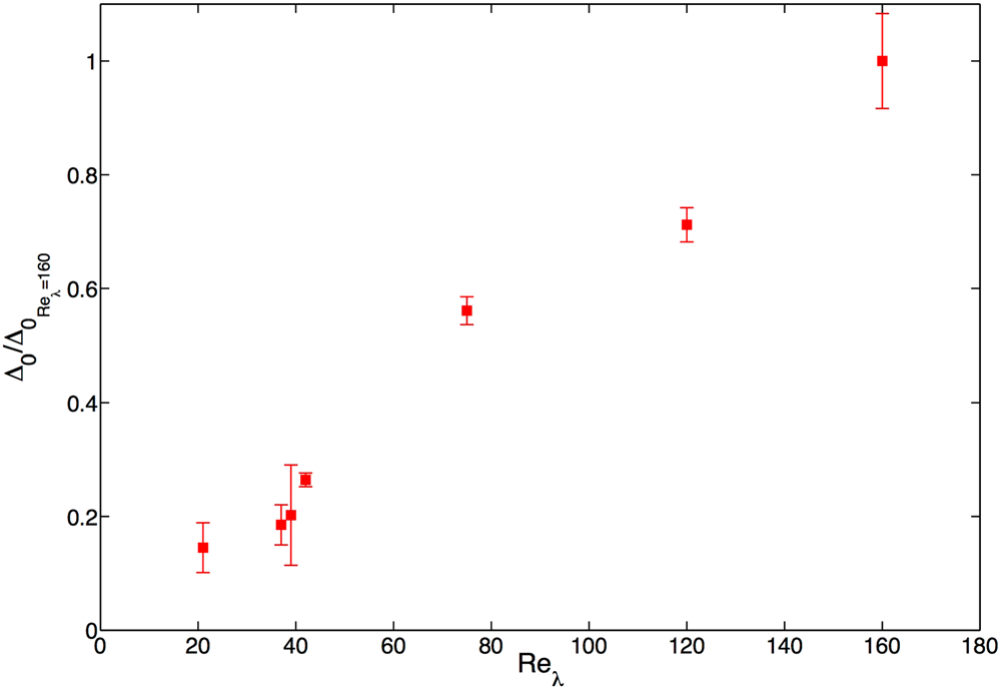
Effect of the Reynolds number. A plot of the ${{\rm{\Delta }}}_{0}/{{\rm{\Delta }}}_{{0}_{{R}{{e}}_{\lambda }=160}}$, from our 3D simulations, for various values of *Re*_*λ*_.

At this stage, before we turn to a discussion of our results, it is important to remark about the constant of integration used to obtain the impact velocities. **Δ**_0_ is the typically velocity difference with which two droplets come near each other. From very general conditions, it is likely that **Δ**_0_ should depend on turbulent intensity, the spatial dimension, as well as the relative Stokes numbers of the fluid (when $S{t}_{1}$, $S{t}_{2}\sim {\mathscr{O}}\mathrm{(1)}$. However we do not have an analytical expression for **Δ**_0_ and as our theoretical and numerical results suggest, **Δ**_0_ is likely to be a constant or an algebraic, sub-dominant prefactor at least in the range of Stokes numbers studied here (For droplets which are large, it is plausible that **Δ**_0_ ~ *St* ^25^). From our data, we are however, able to extract the Reynolds number dependence of **Δ**_0_. In Fig. 5, we show a plot of a normalised **Δ**_0_ versus *Re*_*λ*_ from our simulations in three dimensions; not surprisingly we find an increase in **Δ**_0_ as the system becomes more and more turbulent.

It is important to stress the rationale behind using two-dimensional flows (and neglecting coalescences) when we measured our enhancement factor in the collision frequency. There have been a lot of studies which show that very often the correct framework to study such geophysical or astrophysical systems is the two-dimensional Navier-Stokes equations or its quasi-2D variants^34^. Besides, this *exploratory*, theoretical work underlines the importance of the often neglected transients in such non-equilibrium processes. Consequently, the dimensionality of the flow does not detract from the qualitative message while at the same time the relative computational ease of two-dimensional simulations allow us to obtain more statistics and hence a firmer confirmation of our theoretical conjectures.

Before concluding, it is worthwhile to note that our work is two-fold and aimed at contributing to our understanding of turbulent transport and its role in forming larger aggregates through coalescence. A stumbling block in a fully self-consistent theory to explain such accelerated growths is the lack of realistic collision-coalescence kernels for different-sized particles which incorporate both fragmentation and coalescence. A first step in this direction is, of course, determining the dependence of impact velocities of colliding particles on their sizes. Our results, summarised in Table 1, are important in their implication. We show that the larger particles (large Stokes numbers) do not collide with arbitrarily large velocities with the smaller, tracer-like particles but actually saturate (~exp(−1/*St*)). This suggests that in inhomogeneous suspensions, such as the polydisperse droplet distribution in warm clouds, a run-away growth for large droplets through coalescence (and not fragmenting because of large velocity differences) is likely to be the dominant mechanism triggering rain^22,23^. Remarkably, also, our results seem to be independent of dimension. It should be noted that similar results can be obtained from the calculations of collision rates in simplified statistical models as shown, e.g., in Refs.^8^^,^^16^ when the colliding particles have the same Stokes numbers. In particular we have shown that there is a limiting form for the impact velocity and hence in natural settings coalescence–and not fragmentation due to large **Δ**–should be the dominant mechanism. Therefore this work is a significant step in developing models for coalescing droplets. Important questions related to Reynolds and Froude number effects is beyond the scope of the present work and the issue of collision frequencies in polydisperse suspensions is addressed elsewhere.

The second aspect of this work has to do with collision frequencies and the possible enhancement of collisions in the transient phase (as defined before). In this part, we ignored real coalescences or collisions. This is because in we wish to understand whether transients–which are ignored in theory and simulations but present in nature–lead to an enhancement in the rate at which different-sized droplets collide. A fraction of such collisions lead to coalescences. So our focus has been in understanding where enhanced collisions can arise from which in turn lead to rapid growth in droplet formation. Coalescence is important, at this level, to the extent that it leads to poly-dispersity. Hence although, as we explained, coalescences can be ignored (to begin with) the effect of poly-dispersity cannot. This is why in our study we do include poly-dispersity but not coalescence or collisions. We refer the reader to Bec, *et al*.^22^ for a detailed study of coalescences and droplet growths in a turbulent flow in the statistically steady regime.

We thus addressed the neglected question of how particles evolve in the transient phase through a systematic numerical study of heavy inertial particles in a turbulent flow. We find the remarkably striking result that in the transient, evolving phase, collisions between dissimilar particles are enhanced by more than a factor of two as compared to the rates in steady states. Previous studies have typically concentrated on non-equilibrium steady states as well as mono-disperse suspension. And it is within that framework, that earlier studies have tried to understand the observed fast time scales in which droplets grow in a suspension advected by a turbulent flow. Our work, which opens up an entirely new possibility, suggests that the answer to explain rapid growth of droplets may well lie in the dynamics of particles in transient phases where increased collision rates between large and small particles result in runaway process and a rapid increase in the number of large droplets. We have reported results for the collision frequencies from our two-dimensional study but preliminary results suggest that the phenomena is valid in three dimensions as well. However we will be carrying out a systematic study in three dimensions which will be reported elsewhere. Furthermore, given the results on gravitational settling for heavy particles^21^, it would also be important to study the effect of gravity on these transient collisions in three-dimensional flows. We note that for these results reported here, we have not included gravitational effects.

Our work, given its premise, throws up several interesting and open questions. It is now important to investigate systematically this enhancement in the case of three-dimensional turbulence and to observe them in controlled laboratory experiments. Furthermore, the effect of transients in the flow itself need to be studied in great detail, with different classes of initial conditions, with collisions and coalescences, as well as possible Reynolds number dependence on the effect of enhancement. Also, given the rather novel framework proposed in this work for accelerated droplet growths, it would be important to test this hypothesis in systems which allow a two-way coupling between the particles and the fluid, as well as, to test the key idea in this work on two-dimensional synthetic flows by using the different approaches^35,36^ to see if we can obtain a theoretical estimate of our enhancement factor. Finally, a key to having a microscopic understanding of particle dynamics, inter alia coalescence rates, may well lie in having a quantitative theory for how the dynamics relax to its stationary distribution as illustrated in Fig. 3(b). These questions will be addressed in future work

## Methods

We perform direct numerical simulations (DNSs) of Eq. (3) coupled with either Eq. (1) or Eq. (2). We solve Eq. (1) (Eq. (2)) on a 2*π* periodic domain, with *N* ^3^ (*N* ^2^) collocation points, by using a standard pseudospectral method and a second-order Runge-Kutta scheme for time-marching. We drive the system to a statistically steady non-equilibrium homogeneous, isotropic turbulent state via the large-scale forcing **f** at small wavenumbers. We perform simulations involving particles with 200 different Stokes numbers and the representative results reported here were obtained with simulations upto a million particles. We use a bilinear interpolation technique to obtain the fluid velocity ***u***(***x***_i_) at the (typically off-grid) particle positions whilst solving for Eq. (3). We list the various parameters of our simulation in Table 2.

**Table 2:** Parameters for our simulations.

Dimension	*N*	*N*_*p*_	*ν*	*k*_inj_	*η*	*k*_max_ *η*	*λ*	*Re*_*λ*_	*τ*_*η*_ (*τ*_min_)
3D	512	10^6^	0.001	1 and 2	0.0059	1.01	0.0856	121	0.0351
2D	1024	10^5^	10^−5^	4	0.0044	1.50	0.20	440	1.92

*N* is the number of grid points along each direction, *N*_*p*_ is the number of Lagrangian and heavy inertial particles, *ν* the kinematic viscosity, *ε* is the fixed energy input, *k*_inj_ the forcing wavenumber, *η* ≡ (*ν* ^3^/*ε*)^1/4^ the dissipation scale, $\lambda \equiv \sqrt{\nu E/\varepsilon }$ the Taylor microscale, *Re*_*λ*_ ≡ *u*_rms_ *λ*/*ν* the Taylor-microscale Reynolds number, and ${\tau }_{\eta }\equiv \sqrt{\nu /\varepsilon }$ the Kolmogorov time scale. The analogous definiton for 2D flows is given in the text.

Let us now describe how we characterise the collision rate *R* in the transient phase or the collision rate *R*_∞_ in the stationary regime. By definition *R* and *R*_∞_ are functions of the Stokes numbers *St*_1_ and *St*_2_ of the colliding pair. If we denote the position vectors of particles ① and ②, of radius *a*_1_ and *a*_2_, by **r**_1_ and **r**_2_, then a collision is registered when |**r**_1_ − **r**_2_| = *a*_1_ + *a*_2_. The collision rates *R* and *R*_∞_ is defined as the number of collisions between particles ① and ② per unit time and normalised by the total number of particles *N*_*p*_ in the suspension. At long times $T\gg {\tau }_{{\rm{\min }}}$, the particle dynamics settle to a non-equilibrium stationary distribution. In this stationary regime, we compute the number of collisions *N*_*c*_ between ① and ② over a time interval ${\rm{\Delta }}t\sim {\mathscr{O}}\mathrm{(1000}{\tau }_{{\rm{\min }}})$ and thence obtain ${R}_{\infty }=\frac{{N}_{c}}{{\rm{\Delta }}t{N}_{p}^{2}}$. At shorter times $t\ll T$, when the dynamics are in a transient phase, we calculate the number of collisions *N*_*c*_ between ① and ②. Since this is a transient phase, we do not average over time (as we do for *R*_∞_) but average over several independent initial configurations *N*_config_ of the particles. We therefore obtain $R=\frac{{{\rm{\Sigma }}}_{{N}_{{\rm{config}}}}{N}_{c}}{{N}_{{\rm{config}}}{N}_{p}^{2}}$ corresponding to time *t* (Collision detection is an *O* (${N}_{p}^{2}$) problem and, hence, is computationally prohibitive for large values of *N*_*p*_. We thus use a grid based algorithm where the 2*π* × 2*π* domain was divided into grids such that no particle crosses more than one grid during one time step. For each particle, collisions with other particles in the same grid and adjacent grids were counted. This procedure ensures that no collision is missed). From our definition, it is obvious that *R*, unlike *R*_∞_, depends on time and as *t* → *T*, *R* → *R*_∞_. In our study of transient phases, we perform simulations with several initial particle configurations *N*_config_. Each initial configuration consists of particles distributed randomly in the flow with zero velocity; different initial conditions thus differ from each other in the initial particle positions. Our results for various sets of initial conditions were in agreement with each other within the error-bars (which are of the order of the symbol sizes in the figures shown).
